# Declining Prevalence of Methicillin-Resistant *Staphylococcus aureus* Septic Arthritis and Osteomyelitis in Children: Implications for Treatment

**DOI:** 10.3390/antibiotics9030101

**Published:** 2020-02-28

**Authors:** Lindsay Weiss, Amanda Lansell, Janet Figueroa, Parminder S. Suchdev, Anjali Kirpalani

**Affiliations:** 1 Division of Hospital Medicine, Joe DiMaggio Children’s Hospital, Hollywood, FL 33021, USA; Liweiss@mhs.net; 2 Pediatric Hospital Medicine, Rainbow Babies & Children’s Hospital, Cleveland, OH 44106, USA; amanda.lansell@uhhospitals.org; 3 Department of Pediatrics, Emory University School of Medicine, Atlanta, GA 30322, USA; Janet.figueroa@emory.edu (J.F.); psuchdev@emory.edu (P.S.S.); 4 Division of Hospital Medicine, Children’s Healthcare of Atlanta, Atlanta, GA 30322, USA

**Keywords:** osteoarticular, bone and joint, microbiology, epidemiology, MRSA, MSSA, resistance trends, antimicrobial stewardship

## Abstract

This study sought to assess clinical characteristics and differences in outcomes between children with Methicillin-resistant *Staphylococcus aureus* (MRSA) and Methicillin-sensitive *Staphylococcus aureus* (MSSA) osteomyelitis or septic arthritis and whether initial antibiotic regimen affects patient outcomes. We analyzed records of children ages 15 days to 18 years admitted between 2009 and 2016 to two tertiary children’s hospitals who were diagnosed with an osteoarticular infection and had a microorganism identified. A total of 584 patients met inclusion criteria, of which 365 (62.5%) had a microbiological diagnosis. MSSA was the most common pathogen identified (45.5%), followed by MRSA (31.2%). Compared to MSSA, patients with MRSA had a higher initial C-reactive protein and longer hospitalization. Patients whose initial antibiotic regimens included vancomycin had a longer hospitalization than those initiated on clindamycin without vancomycin, even after removing sicker patients admitted to the pediatric intensive care unit. While MRSA was associated with increased severity of osteoarticular infections compared to MSSA, the incidence of MRSA has been declining at our institution. Patients with longer lengths of stay were more likely to be on vancomycin. Clindamycin should be considered in the initial antibiotic regimen for osteomyelitis and septic arthritis with ongoing surveillance of local microbiology and outcomes.

## 1. Introduction

Acute hematogenous osteomyelitis (AHO) and septic arthritis (SA) are frequent causes of hospitalization in the pediatric population and can have significant morbidity if not quickly recognized and treated appropriately [1,2,3]. The bacteria most commonly isolated is *Staphylococcus aureus*, which accounts for greater than 50% of culture positive cases [1,4,5,6].

Over the past two decades, musculoskeletal infections caused by Methicillin-resistant *Staphylococcus aureus* (MRSA) have been increasing, whereas the rate of Methicillin-sensitive *Staphylococcus aureus* (MSSA) infections has generally remained steady [7,8,9,10]. MRSA osteoarticular infections are frequently associated with higher inflammatory markers [8,11,12], a higher complication rate [7,8,13], longer lengths of hospitalization [8,12,13], and higher associated hospital costs [13]. However, more recently studies have shown a decrease in the proportion of *S. aureus* infections caused by MRSA [14,15,16,17].

The changing epidemiology of these infections in some areas may necessitate a change in empiric antibiotic therapies. The Infectious Diseases Society of America recommends intravenous vancomycin for the treatment of children with acute hematogenous MRSA osteomyelitis and septic arthritis; however, they mention that clindamycin can be used as empiric therapy if the clindamycin resistance rate is low (e.g., <10%) and the patient is stable and does not have evidence of ongoing bacteremia or intravascular infection [18]. In a review by Harik and Smeltzer, the authors recommend use of clindamycin if community-acquired rates of MRSA are >10% and clindamycin resistance is ≤25% [19]. McBride et al. found that in children with AHO, hospital readmissions rates and 72-hour improvement in C-reactive protein (CRP) values were similar between patients who received early treatment with MRSA coverage, compared to MSSA-only coverage. However, they did not examine differences between initial MRSA drug choices (e.g., clindamycin versus vancomycin) on patient outcomes [20]. Few studies have examined clinical outcomes based on empiric therapy for AHO or SA. 

This study aimed to describe the microbiological and clinical characteristics of AHO and SA in a large cohort of children and to compare outcomes between MSSA and MRSA. A secondary aim was to examine the outcomes of children treated initially with vancomycin-containing regimens, compared to those treated with clindamycin without vancomycin. 

## 2. Methods 

### 2.1. Study Design and Participants

A large retrospective cohort study was performed on children ages 15 days to 18 years admitted to two tertiary care children’s hospitals affiliated with Children’s Healthcare of Atlanta between 1 January 2009 and 31 December 2016 with a diagnosis of SA and/or AHO. Details on the overall study design and methodology have been previously reported [21]. In brief, patients were identified through a retrospective search of electronic medical records using International Classification of Diseases, Ninth Revision (ICD-9) and International Classification of Diseases, and Tenth Revision (ICD-10) diagnostic codes for osteomyelitis and septic arthritis. The medical records of all patients who met initial inclusion criteria were then manually reviewed. We excluded the following: any patient who had not been treated for osteomyelitis and/or septic arthritis; had recent trauma, including fractures or foreign body, a postoperative infection, or hardware at the site of the infection; had chronic symptoms (>6 weeks in duration) or had been diagnosed with chronic recurrent multifocal osteomyelitis; were immunocompromised; were a neonate who had never left the hospital; or had had the majority of their workup performed at an outside hospital. The study protocol was approved by the Children’s Healthcare of Atlanta Institutional Review Board. 

### 2.2. Data Collected

Demographic features, type of infection (osteomyelitis, septic arthritis, or both), duration of symptoms at presentation, initial white blood cell count, inflammatory markers, fever at presentation, location of infection, microbiological results, and initial antibiotic regimen were, retrospectively, reviewed and analyzed. 

For our primary variable of interest, we analyzed microbiological data from cultures to determine if patients were infected by MRSA, MSSA, other organisms (non-*S. aureus*), or if no pathogen was able to be identified. We focused on the differences between MRSA and MSSA, since these are the most frequently identified organisms. Positive cultures were identified from samples collected from blood, synovial fluid, bone, or wounds/abscesses. Our primary outcomes of interest were length of hospitalization and readmission within 60 days for a problem associated with initial hospitalization for AHO or SA. Our secondary outcomes of interest were patients requiring a pediatric intensive care unit (PICU) admission and those found to have a subperiosteal abscess. We also examined clindamycin sensitivity rates for all patients who had an infection with *S. aureus*. Clindamycin susceptibility was defined as standard laboratory susceptibility as well as the absence of inducible resistance to clindamycin when this was tested. Isolates with intermediate susceptibility to clindamycin were considered resistant in our study. 

A tertiary variable of interest was comparing patients who were initially placed on an empiric antibiotic regimen containing clindamycin (without vancomycin) to those who were placed on a regimen containing vancomycin. Clinical features and patient outcomes were then compared for these two antibiotic groups. An exploratory analysis was also done looking at the effect of initial antibiotic discordance for clindamycin-resistant and clindamycin-sensitive *S. aureus* on complication rate and length of hospitalization. Antibiotic discordance was defined as when a patient’s initial antibiotic regimen did not include another antibiotic to which the organism was sensitive to.

### 2.3. Statistical Analysis 

Statistical analysis was performed using SAS version 9.4 (SAS Institute, Inc, Cary, NC). Descriptive statistics were used to describe the cohorts using medians and interquartile ranges (IQR) for continuous variables and frequencies and percentages for categorical variables. Categorical variables were compared using Chi-squared tests or Fisher’s Exact tests if cell counts were <5. Continuous variables were compared using nonparametric Wilcoxon rank-sums tests. Demographic and clinical characteristics were compared based on (1) pathogen isolated (MRSA versus MSSA), (2) patients who had clindamycin-sensitive *S. aureus* and those with clindamycin-resistant *S. aureus*, and (3) stratification by initial antibiotic regimen (those treated with clindamycin without addition of vancomycin versus those treated with regimens that included vancomycin). The length of stay outcome was log-transformed due to non-normality in residuals and presented as a ratio of geometric least-squares (LS) means with 95% confidence intervals. Logistic regression models were run for PICU admissions and readmission within 60 days. Results were presented as odds ratios (OR) with 95% confidence intervals. One-sided Cochran–Armitage trend tests were also performed to compare proportions of MSSA and MRSA isolates during 2009-2016 and to determine changes in clindamycin antibiotic susceptibility patterns. A *p*-value of <0.05 was considered statistically significant. 

## 3. Results 

### 3.1. Microbiology of Infections

**Figure 1:**
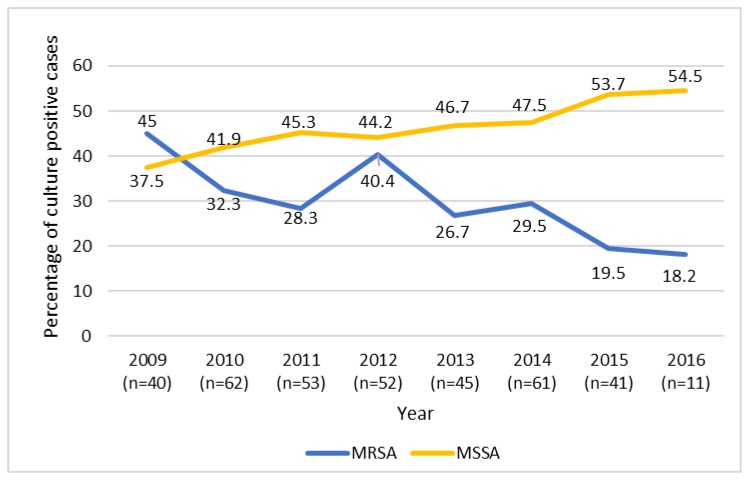
Percentage of Methicillin-resistant *Staphylococcus aureus* and Methicillin-susceptible *Staphylococcus aureus* septic arthritis and osteomyelitis cases out of total culture positive cases by year. Cochran–Armitage trend test for Methicillin-resistant *Staphylococcus aureus* (MRSA), Z-statistic = −2.2876, one-sided *p* = 0.0111. Cochran–Armitage trend test for Methicillin-sensitive *Staphylococcus aureus* (MSSA), Z-statistic = +1.6523, and one-sided *p* = 0.0492. *n* = number of culture positive cases.

A total of 584 patients met inclusion criteria of which a pathogen was isolated in 365 patients (62.5%). The most common pathogen identified was MSSA (45.5%), followed in decreasing frequency by MRSA (31.2%), *Streptococcus pyogenes* (6.6%), *Streptococcus pneumoniae* (3.8%), *Streptococcus agalactiae* (3.0%), other streptococcal species (2.7%), and *Kingella kingae* (2.2%). The remaining 5% of isolated pathogens individually accounted for <1.5% of culture-positive cases and included *Salmonella* species, coagulase-negative staphylococci, *Hemophilus influenza*, *Enterobacter aerogenes*, *Fusobacterium* species, *Bacteroides fragilis*, *Corynebacterium striatum*, *Pseudomonas aeruginosa*, *Citrobacter freundii*, *Propionibacterium acnes*, *Neisseria flavescens*, and *Candida parapsilosis*. *S. aureus* was found in 60.5% of patients with AHO, but only 17.6% of patients with SA (*p* < 0.05). Figure 1 displays percentages of MSSA and MRSA culture-positive infections from 2009 to 2016. During the 8-year study period, the percentage of culture positive cases due to MSSA increased from 35.9% in 2009 to 54.5% in 2016 (z = +1.65, *p* < 0.05). The reverse trend was seen for MRSA infections; 43.6% of culture positive cases in 2009 were caused by MRSA, down to 18.2% of culture positive cases in 2016 (Z = −2.29, *p* < 0.05). 

The characteristics of patients with MRSA versus MSSA are shown in Table 1. Patients with MRSA were younger; more likely to be African American; more likely to have a concomitant AHO and SA; and had a significantly higher initial CRP, erythrocyte sedimentation rate (ESR), and white blood cell (WBC) count (*p* < 0.05). Patients with MRSA had a longer length of hospitalization, were more likely to require a PICU admission, and were more likely to have a subperiosteal abscess compared to patients with MSSA (*p* < 0.05). However, after adjusting for potential confounders (age, race, infection type, and CRP), compared to patients with MSSA, MRSA patients had a longer length of stay but there were no statistically significant differences in rates of PICU admissions or subperiosteal abscess between these two groups (See Table 2). Table 3 shows adjusted outcomes between patients with MRSA, MSSA, other organisms, and no pathogen identified. The odds of having a subperiosteal abscess were 6.4 times higher for patients with MRSA and 5.1 times for patients with MSSA than for patients that had no pathogen identified (*p* < 0.05). Of the 29 patients readmitted within 60 days, there were no significant differences in readmission rate based on microorganism. 

### 3.2. Antimicrobial Regimen and Susceptibility

In terms of antibiotic susceptibility, 91.2% (103/113) of MRSA isolates, 85.9% (140/163) of MSSA isolates, and 88.0% (243/276) of *S. aureus* isolates (MSSA and MRSA combined) were sensitive to clindamycin. There was one case of MRSA and three cases of MSSA that did not have sensitivity to clindamycin reported. Of the 33 cases of clindamycin-resistant *S. aureus*, 63.6% (21/33) exhibited inducible resistance. There was no significant difference in the rates of inducible clindamycin resistance of MSSA compared to MRSA (*p* = 0.10). During the study period, there was a trend of decreased clindamycin sensitivity for MRSA over time (z = −1.94, *p* = 0.03), but no significant trend was seen for clindamycin sensitivity of MSSA (z = +0.64, *p* = 0.26) or for overall rates of clindamycin sensitive *S. aureus* by year (z = −0.70, *p* = 0.24). 

Empiric treatment in our study most commonly included clindamycin (57.4%), ceftriaxone (51.9%), and/or vancomycin (42.1%). Clindamycin and vancomycin were used together in 56 patients (9.6%). Table 4 shows patient characteristics and outcomes based on initial antibiotic regimen. The adjusted analysis suggests that patients on clindamycin (without vancomycin) had a 16% shorter length of stay than patients on vancomycin-containing regimens, even after removing sicker children who were initially admitted to the PICU and after adjusting for type of infection, CRP, days of symptoms at presentation, presence of MRSA, presence of subperiosteal abscess/deep vein thrombosis/and/or pyomyositis, and those who received surgical drainage (*p* < 0.05) (See Table 5). Only one patient on clindamycin required transfer to the PICU after admission. There were no significant differences in patients with clindamycin-resistant *S. aureus* in terms of the length of stay or in the percentage of patients that required PICU admission regardless of their initial antibiotic regimen of vancomycin or clindamycin without another antibiotic that the organism was sensitive to (results not shown). 

## 4. Discussion 

In this retrospective cohort study, we describe the bacterial pathogens responsible for osteoarticular infections in children at two large pediatric hospitals. Traditionally, MRSA bone and joint infections are considered more severe, leading to greater morbidity than those with MSSA infections [8,12,15]. We found MRSA infections were associated with higher inflammatory markers and a longer length of hospitalization compared to MSSA infections, but contrary to many other studies, we did not find any significant differences in the percentage of patients with severe disease sequelae such as a subperiosteal abscess after adjusting for potential confounders. An et al. [22] found no significant differences in inflammatory markers or length of hospitalization between MRSA and MSSA infections. Together, these findings could represent an increased virulence of MSSA over time. We did not look for Panton–Valentine leucocidin in our study, which is a virulence factor produced by *S. aureus* and has been associated with a more invasive disease [23,24]. It is most often produced by MRSA strains but has also been recently detected in some MSSA strains associated with more severe infections and worse outcomes [15]. 

MSSA accounted for 35.9% of isolates in our study in 2009 and increased to 54.5% in 2016. We observed a significant trend of yearly increase in the frequency of AHO and SA caused by MSSA every year, except there was a slight decline from 45.3% in 2011 to 44.2% in 2012. In 2016, there were fewer overall cases of AHO and SA included in the study sample compared to prior years. While this could potentially affect the results, cases included were at random, and the overall trend of MSSA cases remained consistent with the previous year’s trends. 

In our study, clindamycin and/or vancomycin were frequently used as part of the initial antibiotic regimen for *S. aureus* coverage. Patients who were initially placed on a regimen containing vancomycin had a longer length of hospitalization than those initially placed on clindamycin even after controlling for severity of infection. Historically, vancomycin has been recommended in the empiric therapy of invasive musculoskeletal infections to cover MRSA; however, more recently, clindamycin has been recommended as a potential alternative in patients who are not critically ill and in areas where clindamycin resistance is low [18,19]. In 2018 at our institution, guidelines for musculoskeletal infections were developed that recommend clindamycin instead of vancomycin for *S. aureus* coverage unless the patient was hemodynamically unstable or required intensive care. Clindamycin has demonstrated excellent tissue penetration, including in bone [25]; is well tolerated [26]; and has been effective in the treatment of invasive infections caused by MSSA and MRSA [27]. However, there are no randomized studies comparing the efficacy of vancomycin to clindamycin in AHO and SA in pediatric patients. 

The majority of MRSA and MSSA isolates at both of our study sites were susceptible to clindamycin; however, over the years there was a trend of reduced sensitivity to clindamycin in our MRSA strains but not with the MSSA strains. The opposite trend was seen in a large surveillance study of *S. aureus* isolates in pediatric patients in the Military Health System, with a decline in MSSA susceptibility to clindamycin from 2005 to 2014, whereas the MRSA susceptibility rates remained stable [16]. Increased use of clindamycin may lead to increased resistance, and thus regular surveillance monitoring of antimicrobial susceptibility will be important to inform future treatment guidelines. 

We did not find any significant differences in our outcome measures for clindamycin-resistant *S. aureus* regardless of the initial antibiotic regimen, which gives support that clindamycin may be a safe and effective treatment in these infections while awaiting sensitivity results. However, it should be noted that the number of patients with clindamycin resistance was relatively small, which limits conclusions that can be drawn regarding the possible safety of the empiric use of clindamycin. Additional studies comparing the initial antibiotic regimens for MRSA and MSSA musculoskeletal infections are needed to help guide therapy. 

### Strengths and Limitations

A key strength of our study was our large sample size with manual chart review, which provided a granularity of detail that many other studies have not. We also recognize several limitations inherent to our retrospective study design. First, our study was conducted at two large institutions in nearby suburban areas and thus our findings may not be generalizable to other locations particularly regarding microbiology and antibiotic susceptibilities. Although the overall sample size was large, small numbers of patients in several subgroup analyses could have resulted in lack of statistical power to detect significant differences. In addition, misclassification of exposures and outcomes serve as a potential source of bias. This was mitigated by using a structured chart review with a predefined data collection form. We only examined associations and cannot make any conclusions on causality, and we cannot account for the effects of unmeasured confounders on our observations. In our comparisons of the initial antibiotic regimen, we did not collect data on changes to the initial antibiotic regimen, or the length or route of treatment that could have affected clinical outcomes. While we controlled for various measures of infection severity, it is possible that sickly looking children were treated initially with a regimen that included vancomycin, which we were not able to control for in our analyses. Therefore, the longer hospital stay may be associated with vancomycin treatment due to the choice of primary vancomycin treatment in ill patients with hemodynamic instability at admission. However, we feel this is unlikely because even after removing sicker patients who were initially admitted to the PICU, we still found significant differences. Despite these limitations, our study provides helpful data that may be used to guide empiric antibiotic choices and inform future studies.

## 5. Conclusions

The present study adds to the knowledge of the differences and changing epidemiology of MRSA and MSSA bone and joint infections and may help guide initial antibiotic selection. Empiric vancomycin may not be routinely indicated for all children, and clindamycin may be a reasonable alternative in communities with similar local pathogens and resistance patterns. Patients should be closely monitored for antibiotic responses and therapy adjusted as appropriate depending on culture results and clinical response. Ongoing surveillance of the changing trends in MRSA and MSSA osteoarticular infections is necessary, and more studies comparing antibiotic regimens will be important to continue to guide optimal treatment recommendations.

## Figures and Tables

**Table 1:** Patient characteristics and outcomes based on pathogen isolated.

Median (IQR) or %	*All* Culture Positive Cases *n* = 365	MRSA *n* = 114	MSSA *n* = 166	*p*-value ^1^
**Patient Characteristics**				
Age, years	7.9 (2.7–11.7)	6.0 (3.2–10.9)	10.7 (6.3–12.6)	**<0.001**
Male Sex	60.6	61.4	65.7	0.47
Race/Ethnicity				**<0.001**
African American	37.8	51.8	24.7	**<0.001**
Asian	4.1	0.9	6.6	**0.031**
Caucasian	50.4	43.0	60.8	**0.003**
Hispanic	1.6	1.8	1.8	1.00
Other/Unknown	6.0	2.6	6.0	0.19
Type of Infection				**0.040**
AHO only	51.2	53.5	59.0	0.36
SA only	18.1	7.0	13.9	0.07
Both AHO and SA	30.7	39.5	27.1	**0.030**
Initial WBC count, × 10^3^/mm^3^	11.4 (8.5–15.4)	12.0 (8.5–16.0)	10.5 (7.7–13.8)	**0.031**
Initial CRP, mg/dL	9.3 (4.3–19.5)	17.3 (6.9–25.0)	7.8 (4.1–17.1)	**<0.001**
Initial ESR, mm/h	47.0 (29.5–66.5)	51.0 (32.0–71.0)	44.0 (28.0–60.0)	**0.048**
Duration of symptoms at presentation, days	4.0 (3.0–7.0)	4.0 (3.0–7.0)	5.0 (3.0–7.0)	0.27
Fever at presentation	86.6	85.0	91.6	0.09
Antibiotic regimen				0.31
Regimen on clindamycin without vancomycin	46.7	41.3	47.7	
On vancomycin ^2^	53.3	58.7	52.4	
**Outcomes**				
Length of stay, days	6.7 (4.8–9.8)	8.3 (5.9–13.5)	6.1 (4.7–8.3)	**<0.001**
Re-admitted ≤60 days	5.8	8.8	3.6	0.07
Required PICU admission	11.8	20.2	9.6	**0.012**
Subperiosteal Abscess	32.3	46.5	29.5	**0.004**

^1^ Wilcoxon-rank sum tests for continuous variables or Fisher’s exact (if cell counts <5) or chi-squared tests for categorical variables comparing MRSA vs. MSSA groups. ^2^ Vancomycin-containing regimen (includes some patients on vancomycin and clindamycin). *All* column (*n* = 365) includes all cases with *S. aureus* (*n* = 280) and non-*S. aureus* infections (*n* = 85). Erythrocyte sedimentation rate (ESR) missing for 49 people, white blood cell (WBC) count missing for one person, C-reactive protein (CRP) missing for three people. IQR: interquartile range (25th–75th percentiles). **Bolded** values indicates a statistically significant difference with a p-value less than 0.05. MRSA-Methicillin-resistant *Staphylococcus aureus*, MSSA-Methicillin-sensitive *Staphylococcus aureus*, AHO-acute hematogenous osteomyelitis, SA-septic arthritis, PICU-pediatric intensive care unit.

**Table 2:** Adjusted differences in selected health outcomes comparing Methicillin-resistant *Staphylococcus aureus* and Methicillin-susceptible *Staphylococcus aureus*.

Outcome	Estimate (95% CI)- Odds Ratio or Ratio of Geometric LS-Means for MRSA (vs. MSSA-ref) ^2^	*p*-value
Length of stay ^1^, days	1.27 (1.09–1.48)	**0.003**
Re-admitted ≤60 days (vs. not)	2.21 (0.65–7.56)	0.21
Required PICU admission (vs. none)	1.34 (0.54–3.33)	0.53
Subperiosteal abscess (vs. none)	1.14 (0.60–2.15)	0.69

^1^ Length of stay was log-transformed due to non-normality. Estimates are back-transformed geometric least-squares (LS) means. ^2^ Ratio of geometric least-squares means after back-transforming (exponentiation). All models were adjusted for age, race, infection type (osteomyelitis only, septic arthritis only, and both), fever at presentation, WBC, and CRP. Ref: Referent category (MSSA). **Bolded** values indicate a statistically significant difference with a p-value less than 0.05. MRSA-Methicillin-resistant *Staphylococcus aureus*, MSSA-Methicillin-sensitive *Staphylococcus aureus*, PICU-pediatric intensive care unit.

**Table 3:** Adjusted differences in selected health outcomes by isolated pathogen.

	Comparison	Estimate	
Length of stay ^1^, days	Pathogen Isolated	**Ratio of Geometric LS-Means (95% CI)**	***p*-value ^2^**
	MRSA (vs. none)	1.35 (1.11–1.65)	<0.001
	MRSA (vs. MSSA)	1.26 (1.05–1.52)	<0.001
	MRSA (vs. non-*S. aureus*)	1.14 (0.91–1.42)	0.43
	MSSA (vs. non-*S. aureus*)	0.90 (0.77–1.05)	0.56
	MSSA (vs. none)	1.07 (0.90–1.27)	0.73
	Non-*S. aureus* (vs. none)	1.19 (0.98–1.44)	0.09
Re-admitted ≤60 days	Pathogen Isolated	**Odds Ratio (95% CI)**	***p*-value ^2^**
	MRSA (vs. none)	1.35 (0.26–7.09)	0.97
	MRSA (vs. MSSA)	2.13 (0.48–9.51)	0.56
	MRSA (vs. non-*S. aureus*)	0.83 (0.16–4.45)	0.99
	MSSA (vs. non-*S. aureus*)	0.39 (0.07–2.27)	0.52
	MSSA (vs. none)	0.63 (0.12–3.36)	0.89
	Non-*S. aureus* (vs. none)	1.62 (0.45–4.98)	0.92
Required PICU admission	Pathogen Isolated	**Odds Ratio (95% CI)**	***p*-value ^2^**
	MRSA (vs. none)	1.53 (0.32–7.32)	0.90
	MRSA (vs. MSSA)	1.31 (0.43–4.01)	0.92
	MRSA (vs. non-*S. aureus*)	1.10 (0.20–5.96)	1.00
	MSSA (vs. non-*S. aureus*)	0.84 (0.15–4.75)	0.99
	MSSA (vs. none)	1.16 (0.26–5.23)	0.99
	Non-*S. aureus* (vs. none)	1.39 (0.20–9.64)	0.97
Subperiosteal abscess	Pathogen Isolated	**Odds Ratio (95% CI)**	***p*-value ^2^**
	MRSA (vs. none)	6.40 (2.09–19.66)	<0.001
	MRSA (vs. MSSA)	1.26 (0.58–2.75)	0.87
	MRSA (vs. non-*S. aureus*)	1.44 (0.52–3.97)	0.80
	MSSA (vs. non-*S. aureus*)	1.14 (0.41–3.18)	0.99
	MSSA (vs. none)	5.08 (1.72–15.05)	<0.001
	Non-*S. aureus* (vs. none)	4.46 (1.29–15.42)	0.011

^1^ Length of stay was log-transformed due to non-normality. Estimates are ratios of back-transformed geometric least-squares (LS) means. ^2^ Tukey–Kramer adjusted for all pairwise comparisons. All models were adjusted for age, race, infection type (osteomyelitis only, septic arthritis only, and both), fever at presentation, WBC count, and infection severity (CRP). **Bolded** values indicate a statistically significant difference with a p-value less than 0.05. MRSA-Methicillin-resistant *Staphylococcus aureus*, MSSA-Methicillin-sensitive *Staphylococcus aureus*, PICU–pediatric intensive care unit, WBC–white blood cell, CRP–C-reactive protein.

**Table 4:** Patient characteristics and outcomes based on initial antibiotic regimen.

N = 525 Total Median (IQR) or %		Regimen Containing Clindamycin w/o Vancomycin *n* = 279	Regimen Containing Vancomycin ^1^ *n* = 246	*p*-value ^2^
**Patient Characteristics**	Age	6.2 (1.7–10.7)	5.9 (2.5–11.3)	0.40
	Male Sex	57.3	64.6	0.09
	Race/Ethnicity			0.95
	African American	33.3	34.1	0.85
	Asian	4.3	4.1	1.00
	Caucasian	52.0	53.3	0.79
	Hispanic	1.1	1.2	1.00
	Other	9.3	7.3	0.43
	Type of infection			**0.004**
	AHO	45.5	43.5	0.64
	SA	34.8	25.2	**0.017**
	Both AHO and SA	19.7	31.3	**0.002**
	Initial WBC count, x 10^3^/mm^3^	12.2 (9.0–15.5)	11.0 (8.8–15.4)	0.28
	Initial CRP, mg/dL	6.0 (2.6–12.6)	8.9 (3.6–20.9)	**<0.001**
	Initial ESR, mm/h	43 (28–65)	48 (26–68)	0.24
	Duration of symptoms at presentation, days	4 (2–6)	4 (3–7)	**0.025**
	Fever at presentation	77.8	79.3	0.68
**Outcomes**	Length of stay, days	5.5 (4.0–7.1)	6.7 (4.9–11.0)	**<0.001**
	Re-admitted ≤60 days	3.6	6.1	0.18

^1^ Vancomycin-containing regimen (includes some patients on vancomycin and clindamycin). ^2^ Wilcoxon rank-sum tests and chi-squared (or if cell counts <5, Fisher’s exact) tests. **Bolded** values indicate a statistically significant difference with a p-value less than 0.05. IQR-interquartile range, AHO-acute hematogenous osteomyelitis, SA-septic arthritis, WBC-white blood cell, ESR-Erythrocyte sedimentation rate, CRP-C-reactive protein.

**Table 5:** Adjusted analysis of outcomes based on initial antibiotic regimen.

**Outcome**	**Predictor**	**Ratio of Geometric LS-Means (95% CI) ^2^**	***p*-value**
Length of stay ^1^, days *	Clindamycin (vs. vancomycin)	0.84 (0.76–0.92)	**<0.001**
		**Odds Ratio (95% CI)**	
Re-admitted ≤60 days	Clindamycin (vs. vancomycin)	0.88 (0.36–2.16)	0.77

^1^ Length of stay was log-transformed due to non-normality. ^2^ Estimates are ratios of back-transformed geometric least-squares (LS) means. All models were adjusted for CRP, duration of symptoms (days), type of infection, presence of MRSA infection, presence of subperiosteal abscess, deep vein thrombosis, pyomyositis, and those who received surgical drainage. * Length of stay remained the same when the model was re-run after removing patients who were admitted to the PICU. **Bolded** values indicate a statistically significant difference with a *p*-value less than 0.05. CRP-C-reactive protein, MRSA-Methicillin-resistant *Staphylococcus aureus*, PICU-pediatric intensive care unit.
